# Mr. Bedingfield's Compendium of Medical Practice, &c.

**Published:** 1816-10

**Authors:** 


### *Mr.* Bedingfield's *Compendium of Medical Practice, &c.*

( Continued from page 218. )

Chap. 24. *Diabetes.*—In this disease, more benefit was derived by the Bristol Physicians, from Dr. Watt's plan than from any other employed at the Infirmary.

*Case.* Mary Phipps, ætat. 52, a small woman, and much emaciated at the time of admission, made daily from six to seven pints of nearly straw-coloured urine, strongly flavoured with saccharine matter. She was confined for some weeks to animal food, and restricted in the use of fluids: but no relief being procured, she was directed to lose from ten to twelve ounces of blood thrice a week. The blood presented the appearances described by Dr. Watt. Crassamentum at first destitute of tenacity, and of a dark colour; after the fourth bleeding, becoming firmer, soon afterwards exhibiting a layer of cupped coagulable lymph upon its surface.

This plan was persisted in for three weeks. The urine gradually diminished in quantity to three pints in twenty-four hours, and of its natural odour and taste. When things were brought to this promising state, she unfortunately drank nearly a pint of undiluted spirit of wine, which was followed by immediate intoxication, apoplexy, and in a few hours, death!

*Sectio cadaveris.* Vessels of the dura and pia mater gorged with blood; small quantity of serum in the lateral ventricles, and at the basis of the brain. Kidnies large and flaccid. In the right, an hydatid the size of a hazel-nut. Mr. B. proposes the topical abstractions of blood from the lumbar region in diabetes. We do not think they would prove very beneficial, since diabetes appears a general rather than a local disease.

Chap. 25. *On the Function of the Kidnies.*—We are sorry that we cannot compliment our author on the ingenuity of his *speculative* labours. He conceives that urine is *not* an excrementitious fluid, but that the purer parts of it are absorbed from the bladder, and "may possibly contribute towards the formation of the tears, the saliva, the perspiration, and several other secretions." 243.—We must say, that nature would, in such case, take a round-about way of forming saliva and other fluids, by first depositing them in the urinary bladder!

Mr. B. saw the kidney and ureter of a patient who died of phthisis. The kidney was free from disease; but the ureter was distended with urine to about three times its natural size. The urine was prevented escaping by an impervious stricture in the ureter. The patient appeared to labour under no disease of the urinary organs. Here, therefore, thinks Mr. B. the urine must have been absorbed; otherwise, the tube would have burst. Mr. Johnson's case of "*hydro-renal distention,*" lately published in this Journal, shews that the kidney itself would rather have enlarged than the ureter burst.

Section V. Chap. 1. *Affections of the Bladder.*

Mr. B. is inclined to think that almost all diseases to which the bladder is liable, arise from suffering it to be frequently in a state of extreme distention.

"If the urine (says he) were discharged whenever it, either by its accumulation or acrimony, excited uneasiness, nuclei for calculous concretions would have no opportunity for forming." 246.

We believe, indeed, that very many affections of this organ are owing to the cause in question. For an irritable state of the inner membrane of the bladder, Mr. B. recommends the frequent application of leeches to the perinæum and pubes; the warm bath; alkaline preparations, (particularly the liq. potassæ in doses of six to twenty drops thrice a-day) and injections of tepid milk and water into the bladder.

Incontinence of urine depending on paralysis of the sphincter muscles, about the neck of the bladder, may sometimes be remedied by the application of blisters to the perinæum, pubes, or sacrum; and by the lytta internally.

Inability to expel the urine, (which appears to depend upon paralysis of the muscular coat of the bladder, while the sphincter muscles retain their natural contractility) requires the same treatment, and the urine ought to be drawn off three or four times a day, by means of a catheter. Mr. B. proposes operating for the stone in females *above the pubes,* and still thinks this the best place for the operation in *either* sex. Very few of the medical community will think with him.

Chap. 2. *On Affections of the Rectum.*—To the improper retention of fæces, Mr. B. attributes, and perhaps with justice, many diseases of the rectum. When excrementitious matters are suffered to remain long in this gut, the watery parts are soon absorbed, and the remaining hardened mass is with difficulty expelled. Hence, during the subsequent straining at stool, the abdominal muscles, by their pressure on the abdominal viscera, propel an undue quantity of blood into the rectum and other pelvic viscera, whereby the coats of the vessels become overdistended, and by degrees weakened and varicose. Hence arise hæmorrhoidal tumours and effusions of blood. Indurated fæces also irritate the rectum, and excite a spasmodic contraction of the sphincter muscles, thereby producing other serious impediments to their expulsion. The treatment, of course, will be guided by these principles, and attention to the calls of nature must be observed. Hæmorrhoids of recent formation may generally be cured by mild aperients, as confection of senna with sulphur. When piles are very painful, leeches and henbane ointment will afford most relief. Strictures of the rectum of course require the bougie.

Mr. B. has met with several cases of diseased rectum, in which an opening into the cavity of the pelvis existed. Through this opening he has drawn lumbrici.

"A fistulous opening of this description, as well as the more common form of fistula in ano, will often be met with in phthisical patients. Fools, mad men, or bad men only, will operate upon such patients."

Chap. 3. *On Affections of the Uterus.*—Under this head Mr. B. places hysteria, though he thinks the brain, rather than the womb, is the real seat of the disease, the uterus only suffering sympathetically.

" A natural desire to possess that which the laws of society forbid, produces an irritability of mind, and a consequent determination of blood to the head; the sympathy between the brain and the generative system, although inexplicable, is well known to be direct." 255.

*Treatment.*

" We shall find nothing so effectual for the *relief* of hysteria as venæsection. In plethoric habits, it is absolutely demanded; its occasional employment is highly necessary." 255.

This doctrine will not tally with that of the practitioners of the old school, or even with the *men-milliner-medicos* of the modern, especially in this great metropolis, where *hysteria* as naturally suggests *hartshorn,* as *weakness* implies the necessity of *bark* or *steel,* and with equal propriety.

" A spare diet and exercise must be directed, and a proper attention paid to the intestinal canal. *The whole class of stimulants so generally employed must be studiously avoided.*" ib.

We know not to whom this advice will give the greater offence—the prescriber or the patient?—But we shrewdly suspect, that it is as beneficial to the health of the *latter,* as it is inimical to the interest of the *former.* Certain it is, that it will be relished by neither!

" A great majority (says Mr. B.) of the cases of hysteria which have presented themselves at this hospital, have been combined with increased action of the heart, inordinate throbbing of the carotid arteries, and not unfrequently with strong pulsations of the aorta, or cœliac artery." 256.

We believe that these symptoms would be more frequently found, were they particularly sought after; and surely they indicate a very different treatment from that which is too often pursued in hysterical diseases.

In *emansio mensium,* Mr. B. has found monthly venæsections, electric shocks applied to the pubic, hypogastric, and lumbar regions, and the administration of aloetics, successful.

The same treatment is recommended in amenorrhœa.—

"Chorea, emansio mensium, and amenorrhœa, are the only affections in which I have ever found electricity of the slightest service." 257.

We think this by far too sweeping an assertion. The observations of our able Correspondent, Mr. Golding, will be a potent draw-back on the non-electric assertions of our author.

### SECTION VI. *Disease of the Spine.*

Mr. Bedingfield is of opinion that disease of the spine does not occur half so often as is supposed. We think, however, that till very lately, it occurred more frequently than was suspected. The public attention, within these few years, has been more forcibly drawn to the subject; and of course, a number of medical sciolists will trace every pain in the back to disease of the spinal column, in the same manner as, since Corvisart's work, every ailment "which flesh is heir to," originates in disease of the *heart!* The *liver* is quite out of fashion, and it will probably soon be forgotten that there is such a viscus in the abdomen. The heart and colon are now in vogue; but we suspect that the *brain* is the principal seat of disease among the fashionable of the profession at the present time.

"A delicate female cannot now complain of pain in the back, but a surgeon is immediately sent for to examine the spine. If, after thumbing it, and striking it with his knuckles from occiput to coccyx for half an hour, one is discovered to be, or has been rendered by the examination more tender than the rest, it is very gravely pronounced to be diseased. Incarceration for three months, or sometimes for three years, is immediately directed, and setons or issues made in the back." 261.

Although this passage is somewhat *a la caricature,* yet we believe it conveys a censure which is sometimes merited. The other extreme, however, is much more common, and all the farrago of medicinal prescriptions is sometimes swallowed for *rheumatism,* &c.; when, from ignorance or negligence, the disease of the spine is overlooked.

*Fracture of the Spine.*—James Palmer, 63 years of age, fell from a load of hay on the evening of the 18th of June, 1812, and struck the back of his neck violently against the ground. When the head was elevated, he complained of excruciating pain, and a slight depression appeared in the integuments, above the spinous process of the fifth cervical vertebra. Mental faculties were not impaired; speech rather indistinct; upper and lower extremities completely paralysed, and destitute of sensation; pulse full and slow, about 50 in the minute. Venæsectio ad ℥xij. Sumat. pulv. cathart. This was late in the evening of the 19th.

20th. Appears something better; articulates more distinctly; pulse 80, and regular; slept part of the night; water drawn off by a catheter; still constipated. Another cathartic every four hours.

21st. As yesterday; no stool.—22d. Pulse 48, and very weak; voice feeble and indistinct; died at nine o'clock at night.

*Sectio cadaveris.* From an incision along the spinous processes of the cerv. vertebræ, much extravasated blood escaped; spinous process of the 4th cerv. vert. (from Atlas) with that portion of the vertebra constituting the back part of the medullary canal, broken off from the body of the vertebra. Its inferior edge compressed the medulla spinalis. The latter was not lacerated. On puncturing its investing membrane, a quantity of serous fluid escaped. Body of the vertebra uninjured.

Mr. B. thinks that the case warrants a conclusion, that an operation would have saved the patient.

### Section VII. *Theory of Fever.*

In this Section we can see little novelty.

"I have never observed fever of any description, without being able to trace a determination of blood to some particular organ. Examination after death, whenever it has been resorted to, has confirmed the observation.—All fevers are inflammatory; the division of them into inflammatory and typhoid is prejudicial; it deters many from employing the proper remedies." 271.

*Typhus.*

"I am of opinion with my friend and *preceptor*, Dr. Clutterbuck, that the seat of genuine typhus is the brain."

Very natural to think as our preceptors do — for a few years at least; but probably twenty years hence Mr. B. will think otherwise.

"In the first approaches of typhus, we shall frequently (says he) be able to prevent the disease from establishing itself, by an emetic. Should this be found insufficient to prevent the formation of a febrile paroxysm, as soon as the hot stage is fully formed, we must take away some blood from the arm, according to the strength of the patient and violence of the symptoms."

Mr. B. judiciously draws our attention to the action of the carotid artery, rather than of the radial, as exhibiting a clearer index of the state of the brain. He believes, as we have often inculcated in this Journal, that the symptoms of *apparent* debility in the earlier stages of fever, are merely—

" — the consequence of an accumulation of blood within the brain, by which the energy of that organ is impaired." 274.— " Instead, therefore, of inducing debility by the abstraction of blood in the early stages of typhus fever, we shall, by its removal, enable the brain to reassume its functions." 275.

The pages of our Journal contain ample proofs of our having long promulgated these opinions.

When the febrile catenation is formed,

" A palliative treatment (as our author says) is all we can employ with advantage. We must prevent an accumulation of fæces, by giving every night, or every second night, five grains of antimonial powder, and five of calomel. This combination will generally procure two or three dark, fœtid stools; by the evacuation of which the patient will be mnch relieved." 275.

*Scarlatina.* Ann Young, a very corpulent woman, ætat. 20, at the time of her application had the face and neck covered with a deep scarlet eruption, the same being in patches on the arms, legs, and various parts of the body. The face was swollen much; lips dark blue; vessels of the tunica conjunctiva injected with purple blood; cutis covering the alæ nasi sphacelated; tongue thickly encrusted with a dark brown fur on its upper surface; on its sides, and various parts of the fauces, aphthæ. Complained of great soreness of the throat; very difficult deglutition; breathing extremely laborious; cough incessant; purulent and frothy mucous expectoration; voice hoarse, indistinct, and disagreeable; pulse soft and natural, 90 in the minute. She had been unwell for a fortnight; but seriously ill a week. Eruption had been on the skin three days. To take five grains of calomel and five of antimonial powder at bed-time, using an acidulated gargle for the throat and aphthæ.

*Next day.* Delirium during the night; legs, arms, neck, and different parts of the body, covered with petechiæ of an unusual size; breathing more laborious; pulse less distinct; blister to the chest. Died in the evening.

*Sectio cadaveris.* Abdominal viscera perfectly healthy; lungs gorged with blood; some serum in the cavity of the chest; lining membrane of the larynx of a dark brown colour, resembling wetted brown paper. The same appearance was seen for a short distance upon the membrane of the œsophagus. The membrane of the trachea in the highest possible state of inflammation, which extended into the bronchia as far as they could be traced, the redness gradually assuming a brighter hue as it extended itself on the bronchia. Brain not examined.

"This patient evidently (says Mr. B.) fell a victim to inflammation of the larynx, trachea, and bronchia, not to scarlatina." 284.

*Rubeola.* Mr. B. justly remarks, that immense numbers of children die annually of measles, merely because it is considered a trifling disease by parents, not requiring medical assistance. When it is prevalent in the neighbourhood, children who have not had it should be prohibited animal food, and otherwise kept on low diet, taking twice a week a gentle aperient. Children thus prepared, would, almost to a certainty, have a mild disease.

*Treatment.* After an emetic in the first stage, venæsection should be employed the *instant* a determination to the lungs is discovered, while leeches are applied to the chest. This is certainly the period of danger, wherein the foundation of all the miserable *sequelæ* of the disease is laid.

"We must take away blood from time to time, so long as difficulty of breathing, a troublesome cough, or any other symptom indicative of inflammatory action in the lungs, exists."

It is of great importance in this disease, that the cough should be kept quiet; and a small quantity of syr pap. alb. swallowed slowly, will effect this better than any other remedy.

*Pertussis.*

"The preparatory measures advised for measles should be resorted to here. Emetics should be given twice a week, or three times; leeches and blisters frequently to the sternum, and along the course of the trachea; and the cough quieted by the judicious use of syrup of poppies. When the inflammatory symptoms run high, venæsection will be requisite. Change of air after a few weeks." 289.

*Syphilis.*

"Mercury is the only certain remedy for syphilis; but I feel persuaded that ten times as much of it is frequently given, as is necessary for the cure of the disease in any form. If we excite and keep up a *slight* mercurial action for a sufficient length of time, the disease will be certainly cured, without any injury to the constitution."

We know not what the practice is in the western parts of the island; but in the eastern and southern, we rarely heard any *regular* practitioner acting otherwise than is described above.

"Unfortunately (says Mr. B.) a syphilitic ulcer in the throat, and an ulcer produced by the excessive use of mercury, bear a very strong resemblance to each other."

They certainly do often resemble each other, and, notwithstanding the plain distinctions laid down by authors, they cannot always be discriminated, as we know from anxious experience.

*Erysipelas.* This disease proved very fatal at the Bristol Infirmary till the *Brunonian* was exchanged for the *Sangrado* practice. Thirty patients fell victims to it! It was the *erysipelas œdematodes,* of which our colleague, Dr. Johnson, has given an account, in the New Med. and Phys. Journal, and Dr. Hutchinson, of Deal Hospital, in the fifth volume of Med. Chir. Trans.

"The brain always exhibits traces of excessive vascular action; its vessels are over-distended with blood; lymph and serum are deposited upon and between the arachnoid coat and dura mater, and at the basis of the brain; and sometimes the lateral ventricles are filled with serum." 299.

*Treatment.*

"Blood was taken largely from the arm, and active purgatives freely administered. Advantage will be derived from keeping up a constant evaporation from the inflamed parts, by means of alcohol and water."

To these directions we would add the utility of *free incisions,* as advised by Messrs. Hutchinson and Johnson, in the works above alluded to.

*Diseases of the Skin.* Here we cannot but think, that Mr. Bedingfield has committed himself by some more *sweeping conclusions.*

"Much more has been written (on diseases of the skin) than is *practically* useful. The *only distinction* which I have found it necessary to make, is between cutaneous eruptions of an *indolent* nature, and those which are *active* or *irritable.* The former will require stimulating applications; the latter, such applications as have a tendency to allay irritation. Mercurial preparations are best adapted for the former; for the latter, nothing will be found more efficacious than an ointment which has henbane for its basis. Sulphur will not only cure scabies, but an immense number of anomalous eruptions. Alteratives are sometimes useful, either by exciting an increased action upon the skin, or by increasing the secretion of the liver." 301.

Now although the distinctions laid down by Willan and Bateman respecting cutaneous defadations cannot be expected to be *all* attended to in the hurry of general practice, or perhaps even by the most discriminating practitioners, yet surely cutaneous diseases are of such importance in their nature, and so various and dissimilar in appearance and treatment, as to require a much more studious arrangement and classification than Mr. Bedingfield has assigned them. In the Charlatan pages of a Wilson we might expect such assertions as the foregoing, but not in those of a man like Mr. Bedingfield.

*Rheumatismus Acutus.*

"Nothing has been found so effectual as copious venæsection. When 20, 30, or 40 ounces of blood have been taken away, for three or four days in succession, cases of the most violent description have been very speedily cured."

Those treated on an opposite plan were slow of recovery, and metastasis to the heart was not unfrequent. Besides venæsection, purgatives were employed; cold applications to the inflamed extremities were sometimes useful. 308.

*Rheumatismus Chronicus.*

"The arsenical solution, the warm bath, and active purgatives, have been found amongst the most powerful remedies for the removal of chronic rheumatism. When these fail, the disease may sometimes be cured by exciting a slight mercurial action in the system.

"Cases, which have long resisted every plan of treatment that could be devised, have at length been cured by giving a *scruple of calomel* twice a week. I never saw any ill effects follow the use of so large a dose of this preparation." 308.

We have now turned over the last page of Mr. Bedingfield's work. By means of our critical sagacity and long sojourn in this world, we perceive that our author is a young man. We hesitate not to say, that he evinces *natural abilities,* which, when farther cultivated by experience, reflection, and study, will one day raise him higher in his profession than he at present stands. His talent for *practical observation* is evidently superior to his genius for theory, and to the *former* we would recommend him to apply, as a surer and more profitable *iter ad astra.*

If our cynical habits, and the moroseness of old age, have betrayed us into any asperity of remark in the early part of our analysis, we here make the *amende honorable.*

We took up the gauntlet of defiance at the beginning; we hold out the olive-branch at the end. With that candour which he himself admires and professes, we have told him his *faults*; but we are not such churls as to draw a veil over his *virtues*. The attention which we have paid to his work, and the pains which we have taken to portray its contents, are the highest compliment which he can ask, or we confer.

